# Mother and Embryo Cross-Communication

**DOI:** 10.3390/genes11040376

**Published:** 2020-03-31

**Authors:** Anna Idelevich, Felipe Vilella

**Affiliations:** 1 Igenomix, Boston, MA 02210, USA; anna.idelevich@igenomix.com; 2 Igenomix Foundation, Instituto de Investigación Sanitaria Hospital Clínico (INCLIVA), 46010 Valencia, Spain; 3 Department of Obstetrics and Gynecology, BIDMC, Harvard University, Boston, MA 02215, USA

**Keywords:** embryo, uterus, window of implantation

## Abstract

Endometrial receptivity is a biosensor for embryo quality, as embryos with reduced developmental potential are rejected. However, embryo quality only accounts for an estimated one-third of implantation failures, with suboptimal endometrial receptivity accounting for the remaining two-thirds. As pregnancy progresses, a uterus continues to engage in close communication with an embryo/fetus, exchanging information in the form of endocrine, paracrine, and other cues. Given the long mammalian gestation period, this dialogue is intricate, diverse, and, currently, not fully understood. Recent progress and the availability of high-throughput techniques, including transcriptomics, proteomics, and metabolomics, has allowed the simultaneous examination of multiple molecular changes, enhancing our knowledge in this area. This review covers the known mechanisms of mother–embryo cross-communication gathered from animal and human studies.

## 1. Introduction

Infertility is common, with ~12% of women in the United States being unable to conceive [1]. To solve this, patients have turned to in vitro fertilization, with considerable success. According to the European Society of Human Reproduction and Embryology (ESHRE), more than 8 million babies have been born from In vitro fertilisation (IVF) since the world’s first IVF-birth in 1978 [2], and currently ~1.6% of all births in the United States now result from this procedure [3]. The success of IVF, however, can vary depending on the cause of infertility. Almost half of infertile cases occur due to endometriosis, but for others, the etiology is unknown [4] and thus a challenge to treat.

Improving the success rate of IVF requires a better understanding of how the embryo interacts with the uterus throughout pregnancy. Embryonic implantation and development are not possible without continuous molecular dialogue. The mother and embryo exchange signals at all times, from embryonic stem-cell differentiation all the way to implantation, decidualization, placentation, and also parturition, resulting in the birth of offspring [5,6,7,8,9]. 

Pregnancy begins with the union between a haploid (23-chromosome) sperm with a haploid egg, forming a diploid (46-chromosome) single-cell zygote, which continues to undergo mitotic divisions and forms a blastocyst while traveling across the fallopian tube toward the uterus. The blastocyst consists of an inner cell mass and an outer trophectoderm cell layer. During implantation, when the blastocyst adheres to the uterine endometrium, the inner cell mass further differentiates into two cell lineages, the primitive endoderm and the epiblast. The epiblast gives rise to the fetus and the primitive endoderm and trophectoderm give rise to fetal membranes and the placenta, respectively [5,6,10].

Pregnancy relies on blastocyst implantation during a narrow window of uterine receptivity, called the window of implantation; implantation outside this window is associated with spontaneous miscarriages. Uterine sensitivity in mice, is divided into two principal phases: prereceptive (days 1 to 3) and receptive (day 4). The uterine transition to the receptive phase, where blastocysts can implant, requires priming with progesterone hormone (P4) traced on estrogen (E2). Upon closure of this window, the uterus is a hostile milieu, and the blastocyst cannot implant. In humans, the pre-receptive phase occurs after ovulation (7 days, early luteal phase), followed by the receptive (~7–10 days, mid-luteal phase) and nonreceptive (~7–10 to 28–30 days, late-luteal phase) phases until menstruation ensues [5,6,11].

The three main tissue compartments of the uterus—the endometrial epithelium (luminal and glandular) and stroma, and myometrium—support and regulate pregnancy. As the site of blastocyst adhesion, the luminal epithelium is perceived as the crucial site for uterine receptivity and transmits signals to other compartments [12]. Histological aspects of the endometrium when it becomes receptive include: irregular glands with a papillary appearance, migration of vacuoles to a supranuclear position in epithelial cells, edematous uterine stroma, and decidualization [13]. 

The contact between the receptive endometrium and the competent blastocyst involves a series of stages: apposition, adhesion, and invasion, constituting a successful implantation [13]. During the apposition moment, a multitude of small microvilli protrusions (pinopodes) develop on the apical surface of the luminal epithelium. These microvilli form a single flowerlike shape, which appears only during the window of implantation, and subsequently inter-digitate with the blastocyst. Uterodomes are characterized by the presence of cell-adhesion molecules (i.e., integrins [14,15]). The basal and lateral membranes also undergo transformations, specifically at various junctions [12]. Blastocyst adhesion with the luminal epithelium overlaps with the process of decidualization of the stromal cells. The changes in morphology and function governed by the two main ovarian hormones—E2 and P4—accord with the associated molecular changes, including elevated expression of estrogen receptor 1 (ER1) and the progesterone receptor (PR) as well as a multitude of downstream target genes. These and subsequent events, leading to the progression of pregnancy and completing in the birth of an offspring, involve a complex interplay of endocrine, genetic, and other cues. 

Presently, little has been published on the molecular mechanisms that control the dialogue between the early embryo and the mother. Several histological and molecular markers have been identified for endometrial receptivity; however, these await consensus [14,16]. For example, even the presence of uterodomes, characteristic of apposition, is not yet a proven significant biomarker [17,18]. Additionally, preoccupation with one potential biomarker at the expense of exploring others remains a challenge in the field [14,16]. This review summarizes what is known of factors implicated in the communication between the embryo and the mother. We start with presenting several important circulating factors, including hormones, cytokines, chemokines, and extracellular vesicles carrying various signaling components, such as microRNAs, and finish with describing the genetic and epigenetic responses of the uterus and placenta to these circulating factors. 

## 2. Circulating Factors

### 2.1. Endocrine—Hormones

Hormones have pride of place in the hierarchy of primary determinants for embryo-uterine crosstalk. Ovarian E2 and P4 are crucial for a series of events ranging from uterine receptivity to implantation, decidualization, placentation, and finally birth. E2 and P4 govern the chronological transitions between these events, supporting continuous interactions between the mother and the developing baby [6,11]. Both hormones affect a plethora of growth factors, transcription factors, lipid mediators, cytokines, and cell cycle regulators involved in the course of pregnancy [5]. 

The role of hormonal signaling has been studied in many model systems. In mice, a “delayed implantation” model is commonly used to assess signaling during pregnancy [19]. This model uses ovariectomy on day 4 before the preimplantation estrogen surge and continued P4 treatment to induce uterine quiescence while maintaining implantation competency, which is resumed upon estrogen repletion. This indicates the importance of these two hormones in controlling uterine receptivity in mice. Specifically, the expression of Sik-SP regulated by E2 is independent of ER but requires the control of estradiol receptor α (ERα) necessary for the coordination of the biphasic responses in the uterus for its growth [20]. However, while P4 is an absolute requirement for implantation in many species, ovarian estrogen rise is not crucial in subhuman primates [6]. 

In humans, the 28–30-day menstrual cycle begins with menses. The proliferative phase is influenced by rising E2 levels generated from ovarian follicles, which leads to the proliferation of the endometrial epithelium, stroma, and vascular endothelium to regenerate the uterine lining. At midcycle, the gonadotropins: follicle-stimulating hormone (FSH) and luteinizing hormone (LH), induce ovulation on day 14. Subsequently, in the early secretory phase, the endometrium becomes thicker and the corpus luteum forms from the ruptured follicle leading to a P4 upsurge in preparation for implantation. The increase in E2 levels overlaid on P4 define the window of implantation. In the absence of a viable embryo, there is hormone withdrawal and menstruation. The implanting blastocyst secretes chorionic gonadotropin (hCG) to maintain the corpus luteum, and pregnancy ensues [6].

During pregnancy, the expression pattern of ovarian hormones is dynamic and has compartment-specific functions mediated by multiple hormone receptors. Mice lacking Era or both PR isoforms, PR-A and PR-B, are hypoplastic and infertile [21,22]. Just before starts the embryo implantation, PR is expressed in the luminal epithelium. However, at the beginning of embryo implantation, the expression of PR rapidly declines in the luminal epithelium with increased expression in the stroma that persists throughout decidualization [23]. Mice with an epithelial loss of Esr1 show implantation failure and abnormal expression of estrogen related genes. Mice with an epithelial-specific loss of Pgr are unresponsive to P4 treatment and are infertile due to defects in embryo adhesion, stromal cell decidualization, and unrestrained estrogen-induced epithelial cell proliferation [23,24]. 

Mechanistically, the infertility in these mice is attributed to low expression of leukemia inhibitory factor (LIF), and Indian hedgehog (IHH) [25]. IHH is expressed in epithelial cells and interacts in the stroma with its receptors (Patched and Smoothened), producing the proliferation of the stromal cell. LIF is a cytokine, member of the interleukin-6 family essential for endometrial receptivity and implantation. LIF binds to their LIF receptor (LIFR) that, in partnership with the co-receptor GP130, transduces signals through the signal transducer and activator of transcription 3 (STAT3). Genetic deletion of *GL130* or *STAT3* result in implantation failure [26,27]. Clinically, the role of LIF remains inconclusive. Using a relatively small cohort of hyperstimulated women with diverse etiologies of infertility, administration of LIF did not improve pregnancy outcomes [28]. 

Overall, steroid hormones affect a plethora of downstream factors crucial for pregnancy progression, acting through bidirectional epithelial-stromal communication. HAND2 is one such ovarian hormone-dependent factor thought to be involved in implantation and decidualization. *Hand2* ablation causes infertility, via a mechanism involving upregulation of fibroblast growth factor-extracellular signal regulated kinase (FGF-ERK) signaling [29]. In contrast, the important homeobox transcription factor *MSX1* is less dependent on E2 and P4 levels [30], but may be vital for fertility. Genetic studies in mice suggest that *MSX1* is necessary for embryo implantation, and subsequent studies in humans revealed that the protein levels of MSX1, were significantly reduced in endometrial biopsies obtained of infertile women [31].

Progesterone resistance—a rapidly expanding topic in clinical research—is linked with reduced endometrial receptivity [16,32]. P4 is anti-inflammatory and induces immune tolerance at implantation. Interference with P4 action using antiprogestins, such as RU-486, causes pregnancy loss and infertility [33]. Furthermore, an early rise in P4 reduces the success rate of embryo transfers, even with frozen embryos known to be competent based on subsequent transfers. There is a 2–3-day temporal window of P4 exposure when receptivity is optimal. Overall, data suggests that abnormal P4 exposure or resistance leads to embryo–uterine asynchrony. P4 is also responsible for timely downregulation of ERs, an effect linked to timely expression of integrin αυβ3, which plays a role in blastocyst adhesion to the uterus [16,33]. Clinically, endometriosis has also been associated with progesterone resistance, or irresponsiveness to progesterone signaling, guiding the search for suitable biomarkers underlying this effect [34].

### 2.2. Paracrine—Cytokines, Chemokines

To assess other paracrine factors regulating pregnancy, changes in the level of signaling molecules have been analyzed in maternal blood throughout the course of pregnancy. Using a liquid chip scanning technology, Zhao et al. analyzed 30 circulating factors at 14 time points in pregnant rats [8]. The technology is based on flexible Muti-Analyte Profiling (xMAP), integrating colored microspheres, fluidics, laser technology, and computer programming algorithms. The greatest change in the levels of signaling molecules occurred in the third trimester, with moderate changes in the first trimester, and relatively little changes during the second trimester. During early-pregnancy (days 1–7; first trimester of human pregnancy), the levels of luteinizing hormone (LH) and brain-derived neurotrophic factor (BDNF) were increased and decreased, respectively. In this time frame, sperm–egg binding and fusion occurs, forming the fertilized egg, which moves from the fallopian tube to the uterus and sends stimulatory signals to the endometrium to prepare for blastocyst implantation. Compared with pre-pregnancy levels, the levels of monocyte chemotactic protein 1 (MCP1), interleukin-10 (IL-10), IL-13, and growth-related oncogene (GRO) are elevated at day 5 (equivalent to the second month of human pregnancy). In this window, the so-called “Th2 phenomena” occurs during which maternal T helper 1 (Th1) inhibition and Th2 activation occur, supporting the involvement of the maternal innate and cellular immune response in fetal development and providing mechanisms whereby maternal immune rejection of the fetus is inhibited. However, by day 7 when the fetal heart is fully developed, the reverse occurs. Th2 transforms to Th1 (by the regulation and expression of transcription factors), aiming to activate innate immunity in the embryo.

The shift to mid-pregnancy (days 9–19; second trimester of human pregnancy) results in stabilization of circulating signaling molecules. Growth hormone (GH) and leptin levels increase, promoting muscle growth and fuel anabolism. Th1 and Th2 levels remain stable, indicating adjustment and growth of the fetal immune system and reduction in maternal immune rejection of the fetus, avoiding fetal abortion. Cd4+ regulatory T cells (Tregs) are essential to the maternal immune tolerance, the diminution in number or nonfunctional competence cells are implicated in infertility, miscarriage, preeclampsia and fetal growth restriction [35,36].

During late-pregnancy (days 21–23; third trimester of human pregnancy), IL-2, IL-6, IL-12p70m, IL-18, interferon-g (IFN-g), leptin, and GRO levels increase, while adrenocorticotropic hormone (ACTH) and BDNF levels decrease. At this time, maternal Th1 is rapidly activated, implying immune protection of the mother and fetus in preparation for delivery. Previous studies have also shown that IL-2m, IL-6, and IL18 relate to uterine expansion. Finally, the postpartum period is marked by an increase in vascular endothelial growth factor (VEGF), possibly to repair the wounded tissue, and prolactin (PRL) increases, promoting and maintaining lactation.

Zhao et al. found that over 30 cell types were involved in this intercellular “wireless” communication network and demonstrated common alterations in the level of signaling molecules in maternal serum (such as cytokines, chemokines, and hormones) at various time points throughout pregnancy from pre-implantation to post-delivery in rats. Further investigation of these factors is warranted to evaluate their role in human pregnancy, but separate studies have already identified some paracrine factors in this context. Colony-stimulating factor-1 (*CSF-1*) promotes differentiation of human trophoblast cells into syncytiotrophoblast cells and guide to the production of placental lactogen [37]. Several metalloproteinases have been reported in connection with the invasive ability of the fetal trophoblast, in particular MMP2 and 9 [38,39]. Trophoblastic MMPs are regulated in response to tumor necrosis factor alpha (TNFa), IL-1b, IL-1a, leptin, transforming factor b (TGFb), macrophage colony-stimulating factor (MCSF), and endothelial growth factor (EGF), which are secreted at the fetal–maternal interface from different cells. Endometrial extracellular matrix (ECM) remodeling is essential for successful implantation and placentation and multiple MMPs as well as their substrates are involved in this process. For example, MMP-14 and ADAM10, present in endometrium-derived exosomes, act on IL-8, TGFb, CD44, Notch and its ligand DLL1 promoting their bioactivity [40,41,42,43]. For reference, a recent comprehensive review has summarized the roles of MMPs in embryo–maternal crosstalk [44].

### 2.3. Extracellular Vesicles and Their Cargo—Proteins, Lipids, miRNA

Extracellular vesicles (EVs) were recently shown to play a role in paracrine communication between mother and embryo [9]. EVs comprise a range of membrane enclosed compartments differing in biogenesis, size, and cargo. Their small size facilitates trafficking between local sites. EVs activate surface receptors on target cells, merging with the cell membrane and releasing cargo. EV cargo—proteins, lipids, and genetic material (DNA, RNA, miRNA, and other RNA forms)—reflects the physiological state of the cell of origin and this property has been exploited in the search for biomarkers of various pathologies, including cancer [43,45,46]. 

Based on their origin and size, EVs are generally subdivided into three classes: apoptotic bodies, microvesicles, and exosomes. Apoptotic bodies are the largest EVs (1–5 μm) and form following cytoplasmic membrane blebbing in cells undergoing programmed cell death, or apoptosis. Molecular markers include: phosphatidylserine (PS) (which serves as the “eat me” signal for phagocytes but is also found in healthy cells), thrombospondin, C3b complement protein, VDAC1 (a protein forming ionic channels in the mitochondrial membrane), and calreticulin (an endoplasmic reticulum protein) [9,47]. Microvesicles are 100–1000 nm, and their molecular markers are ADP-ribosylation factor 6 (ARF6), integrins, selectins, and CD40 ligand. Exosomes are small, virus sized particles (30–150 nm) formed by inward budding of the cytoplasmic membrane. Exosomes were long considered to be nanodust, or dust in electron microscopy, but this perception has changed in recent years, with their role evolving from trash cans to biologically active particles [48,49]. Exosomes play known roles in immunomodulation, their most studied function [46,50], and in angiogenesis, thrombosis [51], and pathologies, such as cancer [47]. The molecular markers of exosomes include: CD63, CD9, CD81, ALIX, TSG101, flotillin-1, HSC70, and syntenin-1 [9]. In general, all EVs have biological and pathological roles and act as messengers in cell-to-cell communication. EVs participate in regulating immune responses, in particular triggering the adaptive immune response and suppressing inflammation [52]. Beyond immunomodulation, EVs contribute to synaptic plasticity, deliver neurotransmitter receptors, play a role in tissue regeneration following injury, and modulate cell phenotype [45]. 

EVs have only recently become of interest in the growing field of embryo–mother cross communication. Data has accumulated showing key roles of EVs at preconception from gamete maturation to implantation and throughout pregnancy [53]. Ng et al. first showed that EVs contain a specific subset of miRNAs not detectable in the maternal cells, by the human endometrial epithelial cell-line ECC1 [54]. These EVs were later verified to be present in human uterine fluid. Burns et al. demonstrated that the uterine fluid of pregnant sheep contains EVs positive for CD63 and HSP70 (exosomal markers) as well as small RNAs and miRNAs [55]. Greening et al. demonstrated that the proteome of highly purified exosomes from human endometrial epithelial cells is subject to steroid hormonal regulation by estrogen and progesterone and varies with the menstrual cycle [56]. Villela et al. performed a study showing internalization of miR30d by mouse embryos via the trophectoderm that results in indirect overexpression of adhesion related genes—*Itgb3*, *Itga7* and *Cdh* [57]. In this study, treatment of mouse embryos with miR-30d resulted in increased embryo adhesion [57]. In contrast, the same group also showed that miR-30d deficiency results in reduced implantation rates and impaired fetal growth [58]. Heterogeneous nuclear ribonucleoprotein C1 (hnRNPC1) has also been implicated in the mechanism of cell-to-cell communication [59]. These findings support a model in which maternal endometrial miRNAs act as transcriptomic modifiers of the preimplantation embryo. Analysis of human endometrial liquid biopsy (ELB) material in both natural and hormonal replacement therapy (HRT) cycles revealed a panel of differentially expressed miRNAs, including members of the miR-30 family [60]. Recently, embryos were shown to “talk back” via release of progesterone induced protein (PIBF) packaged in EVs that modulate maternal immune response [7,61]. The presence of EVs in the uterine fluid implies endometrial–embryo cross talk; however, these studies require more thorough exploration.

EV research is rapidly growing, but is still an immature field facing several challenges. Among the notable challenges are a lack of nomenclature for distinct types of EVs based on cellular origin. The terms “microvesicle” and “exosome” have been used mutually in many published manuscripts because of the incomplete understanding of EV biogenesis, inconsistencies and discrepancies in purification protocols, and imprecise characterization [43]. There are many unknowns regarding the biogenesis, route, and function of EVs in reproductive biology. Regardless, EVs are already considered attractive pharmaceutical targets and may be exploited directly as potential therapeutic agents for tissue regeneration and immune response modulation [43]. EVs might additionally be exploited for non-invasive prenatal genetic testing if it is established that embryos transmit EVs carrying genetic material to the mother. 

## 3. Genetic Changes—Receptors, Signaling Molecules

### 3.1. Uterus

The molecular signature of the uterus at the time of implantation shows elevated expression of several factors, but experts agree that currently, none of these biomarkers have been studied in enough detail to validate their usefulness for assessing endometrial receptivity [14]. Regardless, the emerging evidence about their roles in fertility is worth considering.

Mucin 1 (MUC 1) (a highly glycosylated polymorphic mucin-like protein) secreted by the endometrial luminal epithelium is considered a “barrier to implantation”. In humans, MUC1 is expressed in the luteal and pre-implantation phases in a progesterone-dependent manner [62,63,64]. MUC 1 is more abundant in fertile then infertile women [65]. In baboons, MUC1 was also shown to be progesterone- rather than estrogen-dependent, serving as a marker of the pre-implantation phase [65]. Of interest, a recent study [66] investigating similarities between term pregnancy in eutherian mammals and marsupials found that key biomarkers of implantation, including mucin 1, heparin-binding EGF-like factor (HBEGF), and a range or proinflammatory factors, including IL-6, tumor necrosis factor (TNF), and cyclooxygenase 2 (COX2), are consistent between species, suggesting conserved regulation of embryo implantation. There are transcriptome-wide similarities between the implantation in rabbits and humans and the marsupial adhesion process [66]. Specifically, the marsupial study observed that the biomarker osteopontin was consistent in five human microarray studies in relation to the window of implantation [67].

Osteopontin (OPN) is a glycosylated phosphoprotein expressed in the endometrial epithelium and implicated in adhesion and signaling roles at the embryo–epithelium interface [68,69]. OPN is also a bone associated protein, produced by several bone cell types (osteoblasts and osteoclasts) and extra-osseous tissue (skin, kidney and lung). Due to differences in post-translational modification, OPN’s molecule weight ranges from 41 to 75 kDa, and OPN is suggested to have cell type-specific structure and function [70,71]. OPN plays major roles in bone remodeling, inflammation, immune-regulation, and vascularization, as well as in pathologies, including cancer. Several studies have evaluated OPN as a biomarker of tumor progression [70].

Comparative global gene expression studies have demonstrated an increase in OPN in the human endometrium following the LH surge [72], and OPN has been detected in the vicinity of uterodomes and in decidualizing stroma [73]. Moreover, while OPN null mice are fertile, they exhibit reduced pregnancy rates [74]. Mechanistically, OPN interacts with integrins and is classified as a member of the small integrin-binding ligand N-linked glycoprotein (SIBLINGs) family, which includes molecules, such as dentin matrix protein (DMP1), bone sialoprotein (BSP), and others. Binding of OPN to integrins activates their receptors and cytoskeletal proteins, subsequently promoting focal adhesion in the embryo trophectoderm; however, the functional significance of this interaction in endometrial receptivity requires further investigation [75].

Integrins are transmembrane glycoproteins with a and b subunits that mediate several processes, including cell–cell and cell–extracellular matrix (ECM) adhesion. There are integrins expressed constitutively in the luminal epithelium, and others are regulated in a spatial and temporal manner during the menstrual cycle [12,76,77]. Three integrins, A1B1, A4B1, and AVB3, were reported to have unique expression patterns correlating with the window of implantation in women [14,15]. Of these, avb3 is the best characterized. The avb3 integrin emerges on the top of luminal and glandular cell surfaces, coinciding with the aperture of the window of implantation, and its expression continues into pregnancy with expansion of the decidua [33]. The appearance of αυβ3 integrin on the apical surface of the luminal cells is due to its presence in subnuclear secretory granules [33]. Expression of an intact αυβ3 heterodimer is regulated by *HOXA10* transcription factor, whose expression together with αυβ3 is altered in pathologies, including adenomyosis, polycystic ovary syndrome, and endometriosis [14]. However, these observations are opposed by several studies showing no reliable link between integrins and fertility [78,79].

An additional homeobox protein, HOXA11, may have a similar role as HOXA10 in decidualization. Deletion of either protein in mice results in implantation defects [80,81], which are due to uterine (as opposed to embryonic) errors. HOXA10-null mice produce a normal amount of embryos that are capable to implant normally in wild-type surrogate mice; although, wild-type embryos cannot implant in HOXA10-null mice [80,82]. HOXA11−/− mice show a similar phenotype [83]. In the human endometrium, HOXA10 is expressed by stromal and epithelial cells regulated by progesterone in a dependent manner in a menstrual cycle [84]. Precisely how HOXA10 regulates uterine receptivity is unclear, but microarray analysis of murine endometrium transfected with HOXA10 cDNA has identified 40 downstream target genes including clusterin (Clu), phoshoglycerate 3-dehydrogenase (3-Pgdh), and tumor-associated calcium signal transducer 2 (Tacstd2) [85].

### 3.2. Placenta

The placental interface mediates interaction between the mother and fetus, and changes in placental gene expression have been analyzed to examine how it participates in embryo–mother cross-talk [86]. A recent study conducted single-cell transcriptomics on villous tissue from 2 human term placentas, identifying 87 single-cell transcriptomes. Trophoblast cells at term were concluded to be the most abundant cell type. Single-cell gene expression profiles were segregated into five different clusters (three clusters of cytotrophoblast, decidual cells and extravilous trophoblast), based on combinations of known trophoblast markers (KRT7, KRT8, GCM1, CYP19A1) and diagnostic genes with >10 fold higher expression in uterine vs. immune cells. These transcriptomes were grouped into three large clusters of cytotrophoblast cells. The data were further analyzed with respect to two syncytiotrophoblast transcriptomes, collected from a single placenta by laser microdissection as well as the transcriptomes of primary undifferentiated endometrial stromal fibroblast (ESF), and the transcriptome of in vitro primary differentiated decidual cells. Single-cell data was aligned with tissue-level data to estimate cell origin, and the top 25% (2108) most highly expressed genes were found to comprise 80% of the total aligned placental mRNA.

Based on genetic studies in mice, placenta-derived leptin also has an important role in fertility, and is secreted into maternal and fetal circulation produced by the placental trophoblast. Leptin regulates energy metabolism, feeding, and bone. Released from adipose tissue, leptin travels in the circulation until it reaches leptin receptors in the brain, located in hypothalamic nuclei and other sites [87,88,89]. Leptin acts on the hypothalamic–pituitary axis to affect steroid hormone release. An absence of leptin (ob/ob mice) is not lethal but results in early onset obesity, extreme insulin resistance, stunted skeletal and brain growth, compromised immune system, and infertility [90]. Interestingly, fertility can be restored in ob/ob mice by exogenous leptin administration, which is characterized by increased LH and FSH. However, fertility is not restored by food restriction in these mice, suggesting that leptin affects the reproductive system independently of metabolism [91,92]. In pregnant mice and humans, the placenta is a major site of leptin expression [90], but it is unclear what role placenta-derived leptin might play in embryonic development [93]. Leptin receptor expression is abundant in placenta. These receptors may pass information about energy metabolism between the mother and the baby [94].

## 4. Epigenetic and Transcriptomic Changes

Epigenetics is defined as heritable changes in chromosomes without changes in DNA sequence. These changes include histone modification, DNA methylation, and expression of non-coding RNAs. E2 and P4 regulate the expression of their respective receptors, which are important for embryo implantation. Aberrant DNA methylation of the CpG island in the promoter regions of ER or PR has been reported in endometrial carcinoma, suggesting regulation of the uterus by epigenetic mechanisms [95,96]. Factors related to endometrial receptivity, such as *HOXA10* and *MUC1* have also been shown to be controlled by hormone-dependent DNA methylation, which is associated with infertility [97,98], and changes in DNA methylation in the endometrium have been correlated with gene expression during the transition from the pre-receptive to receptive phase in humans [99,100]. Interestingly, comparison of changes in transcriptomes and corresponding DNA methylomes on the same samples revealed association of gene expression and DNA methylation for a number of loci related to endometrial biology [100], suggesting an interplay between hormones and the uterus at the level of the epigenome [101]. In addition, miRNA and circRNA are stably detected in the circulation and EVs. For example, miR-30d is upregulated during the acquiring of receptivity in the endometrium [102], its overexpression induces changes in the transcriptomics and proteomics of the endometrial epithelium [103], and it is involved in the interaction between embryo and uterus, as it is secreted by the uterus and taken up by the embryo [57]. Free hsa-miR-30d and/or exosomes are internalized by embryos through their trophectodermal cells, resulting in overexpression of genes encoding for molecules related to embryonic adhesion (*Itgb3*, *Itga7* and *Cdh5*) [57]. Other miRNA families shown to be important for implantation and that potentially mediate embryo–mother dialog are miR200, Let7, and miR-17-92 clusters [104].

## 5. Conclusions

For a healthy outcome for the mother and baby, a continuous molecular dialog is crucial. The language is based on endocrine, paracrine, and autocrine factors. Despite gathered knowledge, much remains to be discovered regarding how these factors affect genes in the developing organism and how genetic and epigenetic changes are translated into “readable” information. Estrogen, progesterone, and downstream effectors govern differentiation of the stroma and remodeling of the endometrium, making it receptive for embryo implantation. The embryo also sends various signals to the mother, in the form of EVs carrying miRNA and other material, to which the mother responds. The exact routes of communication are not well understood and further exploration is needed. Better understanding of the physiological mechanisms involved in the mother–baby dialogue during pregnancy should allow identification of reliable biomarkers for endometrial receptivity, aiding the treatment of unexplained infertility and increasing healthy birth rates.
